# Trend in admissions, clinical features and outcome of preeclampsia and eclampsia as seen from the intensive care unit of the Douala General Hospital, Cameroon

**DOI:** 10.11604/pamj.2015.21.103.7061

**Published:** 2015-06-09

**Authors:** Eugene Belley Priso, Theophile Nana Njamen, Charlotte Nguefack Tchente, Albert Justin Kana, Tchuenkam Landry, Ulrich Flore Nyaga Tchawa, Romuald Hentchoya, Gerard Beyiha, Marie Patrice Halle, Leopold Aminde, Anastase Dzudie

**Affiliations:** 1 Department of Obstetrics and Gynaecology, Douala General Hospital, Douala, Cameroon; 2 Department of Surgery and Specialties, University of Douala, Douala, Cameroon; 3 General intensive care unit, Douala General Hospital, Douala, Cameroon; 4 Department of Nephrology and Hemodialysis, Douala General Hospital, Douala, Cameroon; 5 Clinical Research Education, Networking and Consultancy (CRENC), Douala, Cameroon; 6 Department of Medicine, Faculty of Health Sciences, University of Cape Town, South Africa; 7 Cardiology unit, Department of Internal Medicine, Douala General Hospital and Faculty of Health Sciences, University of Buea, Buea, Cameroon

**Keywords:** Hypertension, pregnancy, preeclampsia, outcome, intensive care

## Abstract

**Introduction:**

Hypertensive disorders in pregnancy (HDP) are a major cause of maternal morbidity and mortality. We aimed at determining the trends in admission, profiles and outcomes of women admitted for preeclampsia and eclampsia to an intensive care unit (ICU) in Cameroon.

**Methods:**

A retrospective study involving 74 women admitted to the ICU of the Douala General Hospital for severe preeclampsia and eclampsia from January 2007 to December 2014. Clinical profiles and outcome data were obtained from patient records. Statistical analysis was performed using SPSS version 20.

**Results:**

Of the 74 women admitted to ICU (72.5% for eclampsia), mean age was 30.2years and the majority (90.5%) were aged 20-39 years. While overall trend in admission for HDP increased over the years, mortality remained stable. Mean gestational age (GA) on admission was 34.0 weeks (33.5 for preeclampsia vs 35.4 for eclampsia). Most patients presented with complications of which acute kidney injury was most frequent (66.7%). Visual problems were more common in patients with eclampsia compared to preeclampsia (p = 0.01). HELLP syndrome and acute pulmonary oedema (APO) were predominant in patients with preeclampsia, while cerebrovascular accidents (CVA) occurred more in patients with eclampsia. Overall mortality was 24.3%. Presence of APO was associated with mortality in multivariable analysis (O.R.= 0.03, p = 0,01).

**Conclusion:**

Trends in admission for HDP were increasing with high but stable mortality rate. Patients presented late most of whom with complications. Interventions improving antenatal care services and multidisciplinary management approach may improve maternal outcome in patients with HDP.

## Introduction

Hypertensive disorders of pregnancy (HDP) are a major cause of maternal morbidity and mortality [1]. These generally involve hypertension related conditions occurring primarily during pregnancy or may be pre-existing and persist during and or after pregnancy [2]. Generally, HDP are said to complicate 5-10% of pregnancies and account for 10-15% of maternal deaths globally [3, 4]. Recent data suggests that the increasing incidence of HDP is partly due to an increasing trend in obesity worldwide [5, 6]. Furthermore, the resultant worrisome maternal morbidity and mortality from HDP shows some disproportionate affection geographically, as majority of these maternal deaths are found to occur in low and middle income countries (LMICs) [7]. Preeclampsia is a HDP in which there is hypertension (systolic blood pressure (SBP) ≥140mmHg and or diastolic blood pressure (DBP) ≥90mmHg) and proteinuria occurring after 20 weeks gestation, measured on two different occasions at least 4 to 6hours apart in women previously known to be normotensive [8, 9]. In severe preeclampsia, SBP (DBP) is ≥160 (110) mmHg in the presence of proteinuria. When convulsion occurs in the presence of these features, the condition is known as eclampsia [3]. Amongst others, preeclampsia is known to be a disorder of nulliparity, but multi parous pregnant women with new partners have been shown to have a similar elevated risk for development of preeclampsia like nulliparous women [10]. A study in northern Cameroon found teenage status, illiteracy, nulliparity and family or personal history of hypertension as risk factors for HDP [11]. It should be noted that the adverse effects of preeclampsia and eclampsia are not only limited to the mother but also to the foetus with several complications ranging from intra-uterine growth restriction to intra-uterine foetal death [12]. Several theories exist on the pathogenesis of preeclampsia, but at present, it is suggested that the placenta is the primary agent in the development of preeclampsia, hence, removal of the placenta (by termination of the pregnancy is the sole method of treating the condition [13]. Studies continue to suggest the increasing burden of HDP around the world making it a growing public health problem [1, 14]. Currently, data on risk profiles and outcome of preeclampsia and eclampsia are limited in Cameroon. We thus set out to describe the trends in admission, clinical profiles and outcomes of women admitted to ICU for preeclampsia and eclampsia in Douala General Hospital, Cameroon.

## Methods

### Study design, settings and population

A retrospective cohort study conducted from January 2007 to December 2014 in the intensive care unit (ICU) of the Douala General Hospital (DGH). This hospital is a tertiary, referral and teaching hospital located in the economic capital of Cameroon and serves both nationals and patients from other central african countries. During the study period, the number of physicians of both Obstetrics and gynaecology service and ICU increased between 2007 and 2010. These changes are from 04 to 06 consultants for Obstetrician Gynaecologists, from 02 to 06 for Anaesthesiologists, 01 to 04 Cardiologists, and 0 to 02 for Neurologists working at the ICU. Emergency Physicians and nephrologists also took part in the follow-up of admitted patients. Adequately completed medical records of all women admitted to ICU for HDP and who met inclusion criteria for the study were included.

### Study Procedure, variables and data collection

Medical records of women hospitalized to ICU for HDP within study period were sorted and checked for completeness. Using a structured case report form, socio-demographic characteristics like; maternal age, marital status and mode of admission were obtained, obstetric characteristics obtained were; gravidity, parity, gestational age on admission and history or presence of multiple pregnancy. Other clinical characteristics collected were; SBP, DBP, oedema (facial and or pedal), visual problems, neurologic deficit, glasgow coma score (GCS), mode of delivery and type of ventilation (spontaneous or assisted). Biology parameters obtained were; liver and kidney function tests, serum electrolytes (sodium, potassium, chloride), full blood count, prothrombin time. Pregnancy complications and outcomes were; mode of delivery, presence of HELLP syndrome, placenta abruptio, acute pulmonary oedema, cerebrovascular accident, acute kidney injury and death.

### Data analysis

Data was transferred from the case report form to Microsoft excel spread sheet and statistics software; statistical package for social sciences (SPSS) version 20 for cleaning and subsequent analysis. Categorical variables were summarized as frequencies and proportions while continuous variables were summarized as means, standard deviations and median where applicable. Group comparisons were done using chi square and fisher exact tests and students t-test for categorical and continuous variables respectively. Logistic regressions were used to investigate factors associated with mortality. Statistical significance was considered at p < 0.05.

### Ethical Considerations

Ethical and administrative approval was obtained from the Ethics committee of the Douala General Hospital. Being a retrospective study, written informed consent was not required but confidentiality of patient records was also maintained.

## Results

### General Characteristics of Study population

**Figure 1:**
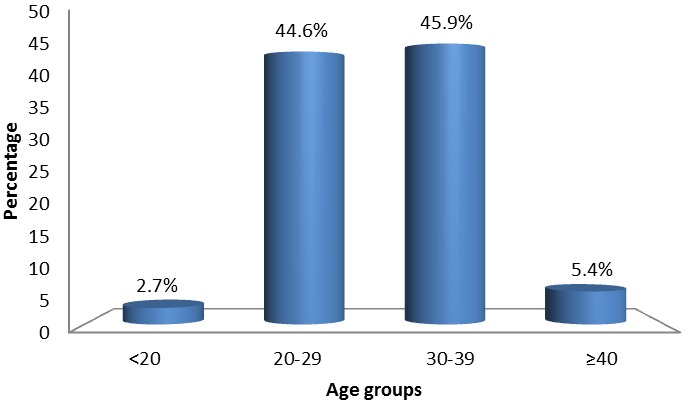
Age distribution of women admitted for preeclampsia and eclampsia in the intensive care unit of the Douala General Hospital from 2007 to 2014

In all, 74 women were admitted to the ICU during the study period (72.5% eclampsia and 27.5% preeclampsia). The overall mean age and mean gestational age of study participants was 30.2 years and 34.0 weeks respectively. Most women were aged 20-39 (90.5%) (Figure 1). Median (range) gravidity and parity was 3 (1-9) and 2 (0-7) respectively. (Table 1) depicts the general characteristics of the study population.

**Table 1:** Characteristics (sociodemographic and clinical) of women admitted for preeclampsia and eclampsia in the intensive care unit of the Douala General Hospital from 2007 to 2014.

General Characteristics	N° missing	Total (n = 74)	Pre-eclampsia [n = 19(27.5%]	Eclampsia [n = 50(72.5]	p-value
**Age, mean±SD (range)**	-	30.2±5.4 (20-44)	30.4±6.0	29.5±4.9	0.53
**Marital Status, n(%)**	19				0.76
Single		21 (38.2)	06 (33.3)	14 (41.2)	
Married		34 (61.8)	12 (66.7)	20 (58.8)	
**Mode of Admission, n (%)**	16				0.17
Domicile		16 (27.6)	08 (44.4)	08 (20.5)	
Referred		33 (56.9)	08 (44.4)	24 (61.5)	
Internal Transfer		09 (15.5)	02 (11.2)	07 (18.0)	
**Gestational Age**,	-	34.0±3.9	33.5±4.6	35.4±3.7	0.13
**Obstetric history**					0.09
Gravidity (median, range)	-	3 (1-9)	2 (1-6)	3.5 (1-9)	
Parity (median, range)	-	2 (0-7)	2 (0-5)	2.0 (0-7)	
Multiple pregnancy, n(%)	-	19 (47.5)	03 (25.0)	15 (55.6)	
**Clinical Characteristics**					
SBP mmHg,	-	160.7±27.3	164.3±31.4	160.0±25.7	0.56
DBP mmHg,	-	101.9±24.6	106.8±26.6	100.6±23.9	0.35
RR,	-	28.0±11.5	36.5±16.5	25.4±9.4	0.07
Temperature, °c	-	37.5±0.9	37.1±0.9	37.6±0.9	0.09
Pedal oedema, n(%)	-	37 (72.5)	09 (69.2)	26 (72.2)	0.83
Facial oedema, n(%)	-	13 (37.1)	03 (33.3)	10 (41.7)	0.66
Coma, n(%)	-	36 (52.2)	08 (44.4)	28 (59.6)	0.40
**Glasgow Coma Score**	6				0.28
13 - 15		34 (50.0)	10 (55.6)	20 (43.5)	
10 - 12		16 (23.5)	06 (33.4)	10 (21.7)	
7 – 9		12 (17.6)	01 (5.5)	11 (23.9)	
3 – 6		06 (8.8)	01 (5.5)	05 (10.9)	
Neurological deficit, n(%)	-	02 (2.7)	00 (0.0)	02 (5.0)	0.36
Visual problems, n(%)	-	07 (30.4)	00 (0.0)	06 (46.2)	**0.01**
**Diuresis**	23				0.92
Normal		22 (43.1)	06 (46.1)	16 (42.1)	
Oliguria		18 (35.3)	04 (30.8)	14 (36.8)	
Anuria		11 (21.6)	03 (23.1)	08 (21.1)	
Transfusion		24 (38.1)	09 (47.4)	15 (35.7)	0.41
**Mode of delivery**	18				
Vaginal		12 (21.8)	03 (17.6)	08 (23.5)	0.63
Caesarean section		44 (78.2)	14 (82.4)	27 (76.5)	0.66
**Type of Ventilation**	12				
Spontaneous		30 (40.5)	10 (62.5)	20 (45.4)	0.24
Assisted		32 (43.3)	07 (41.2)	24 (57.1)	0.26

### Trends in Admission and death

**Figure 2:**
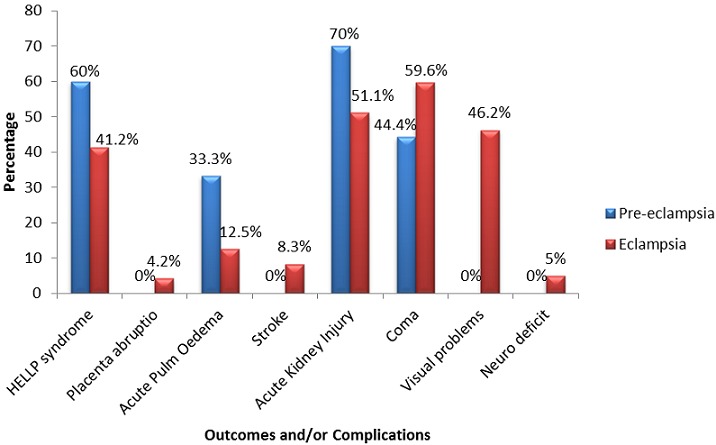
Outcomes and/or complications of women admitted for preeclampsia and eclampsia in the intensive care unit of the Douala General Hospital from 2007 to 2014

Overall, there was an increase in general admissions and admissions for HDP in the ICU between 2007 to 2014. There was an increasing trend in overall mortality in ICU but deaths due to HDP remained stable (Figure 2).

### Complications and Outcome of patients

**Figure 3:**
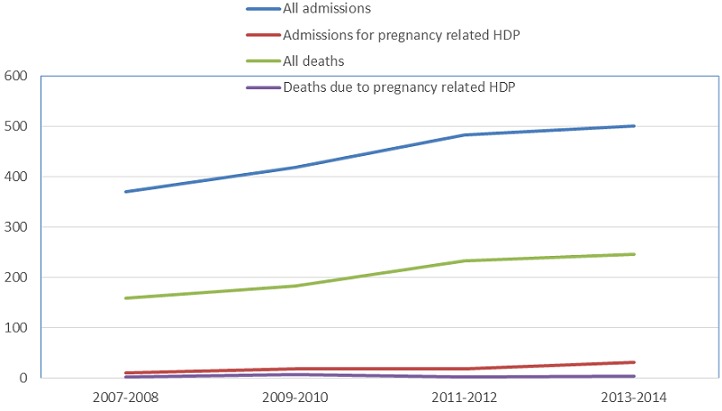
Trend in admissions and deaths due to preeclampsia and eclampsia in the intensive care unit of the Douala General hospital from 2007 to 2014.

Overall median length of hospital stay was 3 (1-10) days and mortality rate was 24.3%. While acute kidney injury (AKI), acute pulmonary oedema (APO) and HELLP syndrome were more common in patients with preeclampsia, (70.0% vs 51.1%, 33.3% vs 12.5% and 60.0% vs 41.2%) respectively, placenta abruptio, cerebrovascular accidents were common in patients with eclampsia (4.2% vs 0.0%, 8.3% vs 0.0%) respectively. Visual problems were more common in patients with eclampsia (p = 0.01), most of whom had assisted ventilation, poorer GCS and more severe leukocytosis (p = 0.01) compared to those with preeclampsia. (Table 2, Figure 3).

**Table 2:** Characteristics (laboratory parameters and complications) of women admitted for preeclampsia and eclampsia in the intensive care unit of the Douala General Hospital from 2007 to 2014

Laboratory parameters	N° missing	Total (n = 74)	Pre-eclampsia [n = 19(27.5%)]	Eclampsia [n = 50(72.5)]	p-value
White cell count,		16,515±7224	12,321.5±4,653.9	18,006.5±7,602	**0.01**
Hemoglobin,		10.7±3.2	10.9±2.7	10.8±3.2	0.95
Hematocrit,		32.5±8.2	32.4±7.4	32.7±8.6	0.91
Platelets		130,879.6±77,683	137,930.7±80,095.9	128,794±78,597	0.72
SGOT,		581.1±1267.6	450.1±888.1	704.8±1459.9	0.63
SGPT,		301.6±586.8	359.9±675.2	318.2±599.2	0.87
TP,		81.8±22.0	80.5±24.2	81.1±21.7	0.94
TCK,		35.2±5.1	35.2±4.7	35.2±5.5	0.99
Urea,		2.4±11.6	7.3±22.9	0.8±0.7	0.10
Creatinine,		32.1±35.2	26.9±33.4	34.1±36.3	0.56
Urine albumin present		13/29 (44.8)	4/9 (44.4)	9/20 (45.0)	0.97
Sodium,		136.6±6.0	133.8±6.1	137.9±5.9	**0.04**
Potassium,		4.7±1.3	4.8±1.7	4.6±1.2	0.65
Chloride		103.3±5.8	101.5±4.6	103.4±6.1	0.34
**Outcome and complications**					
Duration of hospital stay, median(range)days		03 (1-10)	04 (1-7)	03 (1-10)	
HELLP syndrome		20 (45.5)	06 (60.0)	14 (41.2)	0.47
Placenta abruptio		01 (3.3)	00 (0.0)	01 (4.2)	0.61
Acute Pulm. Oedema		06 (18.2)	03 (33.3)	03 (12.5)	0.16
Cerebrovascular Accident		02 (6.7)	00 (0.0)	02 (8.3)	0.46
Acute Kidney Injury		30 (66.7)	07 (70.0)	23 (51.1)	0.84
Deaths		17 (24.3)	05 (26.3)	12 (24.0)	0.70

### Factors associated with Mortality

On bivariate analysis, receiving transfusion [O.R= 4.8 (1.97-24.09), p = 0.04] and presence of APO [O.R = 0.2 (0.01-0.62), p = 0.02] were associated with mortality. In multivariable analysis, APO was the only factor associated with mortality [O.R =0.03 (0.00-0.46), p = 0.01) (Table 3).

**Table 3:** Factors associated with Mortality among women admitted for preeclampsia and eclampsia in the intensive care unit of the Douala General Hospital from 2007 to 2014.

Variable	Bivariate analysis		Multivariate Analysis	
	OR (95% CI)	p-value	OR (95% CI)	p-value
**Age categories**				
20-29	Ref			
30-39	0.3 (0.04-2.83)	0.32		
≥40	0.2 (0.02-2.15)	0.20		
Primigravida	1.2 (0.30-4.53)	0.82		
**Parity**				
Nullipar	Ref			
Primipar	0.9 (0.15-5.86)	0.95		
Multipar	0.8 (0.18-4.04)	0.84		
Multiple pregnancy	0.5 (0.12-2.19)	0.36		
**Gestational Age**				
<30	Ref			
30-36	0.6 (0.05-6.61)	0.65		
≥37	0.5 (0.13-1.97)	0.32		
Fever (Temp > 37.8)	1.3 (0.32-4.83)	0.74		
Pedal oedema	0.3 (0.07-1.83)	0.21		
Facial oedema	3.7 (0.65-21.51)	0.13		
Coma	0.5 (0.15-1.67)	0.26		
**G.C.S categories**				
13-15	Ref			
10-12	0.9 (0.09-10.09)	0.97		
7-9	1.2 (0.09-13.88)	0.91		
≤6	4.2 (0.36-48.44)	0.25		
Convulsion	1.6 (0.45-5.59)	0.48		
**Anaemia**				
Hb ≥ 10g/dl	Ref			
Hb < 10	0.9 (0.22-4.42)	0.98		
**Creatinine**				
<13	Ref			
≥13	0.3 (0.07-1.28)	0.10		
**Urine Albumine**				
Albumin ≤ +	Ref			
Albumin + +	1.6 (0.87-31.87)	0.73		
Albumin + + +	3.3 (0.24-45.10)	0.36		
**Length of Hospital stay**				
**< 7days**	Ref			
**≥ 7days**	0.4 (0.08-1.99)	0.27		
Transfusion	4.8 (1.97-24.09)	**0.04**	8.1 (0.44-133.64)	0.14
HELLP syndrome	0.5 (0.14-2.15)	0.38		
OAP	0.2 (0.01-0.62)	**0.02**	0.03 (0.00-0.46)	**0.01**
Eclampsia	0.3 (0.03-2.52)	0.26		
AKI	0.5 (0.11-1.92)	0.28		

## Discussion

Preeclampsia is a multisystem hypertensive disorder of pregnancy with new onset after 20 weeks gestation which is a leading cause of worldwide maternal and foetal morbi-mortality. This retrospective cohort study conducted in the DGH on HDP revealed an increasing trend in admissions for HDP, high mortality rate from preeclampsia and eclampsia (24.3%) though this was steady from 2007 to 2014. The main complications were AKI (66.7%), HELLP syndrome (45.5%) and acute pulmonary oedema (18.2%) which was the main predictor of mortality. Preeclampsia and eclampsia are invariably not rare affections of pregnancy but this study reports on cases which were severe enough to require admission to ICU. An overall increase in ICU admissions and admissions for HDP over the years 2007 to 2014 was noted in this study. This could be suggested by the growing incidence of hypertension and other cardiovascular related diseases [15] which are the common reasons for ICU admissions. Similarly, high rates of ICU admissions have been reported; Ng et al in Hong kong also reported a double in admission rates to ICU despite improvement in obstetric care [16]. Other studies have reported seemingly increasing rates of ICU utilization and admission rates [17, 18]. The rising ICU admission rates and admissions for HDP could be explained possibly by the re-enforcement and increase staffing in the hospital during the years 2007 to 2010 and changing attitudes of obstetricians and anaesthesiologists in the hospital in a bid to provide optimum care being a referral centre. Moreover, the rising burden of CVDs and hypertension in SSA cannot be overemphasized. Clinical profiles suggest that patients present late with severe complications among which included AKI, visual problems, HELLP syndrome, acute heart failure with pulmonary oedema and even stroke. Some of these complications like AKI are predictors of fatal maternal outcome [19]. Other studies suggest low rates of patients with AKI [1] with current estimates in developed countries as low as 1-2.8% [20]. In developing countries, the incidence of renal failure in HDP is higher with values as high as 36% [21]. In our study, AKI was present in up to 66.7% of cases, further re-iterating the possible late presentation to hospital. Contrary to Seyom and colleagues who reported low rate of cases with renal failure, but rather more frequent was retroplacental haematoma [1]. Acute Pulmonary Oedema was the only predictor of mortality in our study. This was contrary to other reports, where, no antenatal care, high DBP, grand multiparity, high creatinine levels were predictors of maternal death [2, 3, 22].

Overall mortality rate from our study was high (24.3%). This was similar to reports from Ghana [23] and Nigeria [24], though much higher than reported in Ethiopia, where eclampsia related maternal deaths were 11.6% [2]. However, our findings are much lower than reported in a population based study in South Africa which had rates as high as 57% [25] and from India with 67% [26]. We suggest that the high rate of complications resulted in high mortality in our study. Even though this mortality was stable across years, this was probably due to the increase in personnel which probably resulted in better management. The extent of complications overemphasizes on the need of a multidisciplinary team for management, including nephrologist and ophthalmologist. Indeed AKI was found to be more frequent in patients with preeclampsia than eclampsia, which further suggests, that the nephrologist should be brought in at a very early stage so as to limit the proportion of AKI which has been shown elsewhere as a strong predictor of outcome. The retrospective design of the study however limits the study with a number of missing variables which could not obtained from patient records together with the small sample size which didn't permit further exploration of predictors of mortality. However, findings from our study deserve attention, as being the first study conducted in a key referral hospital in central Africa where there is absent data on this worrisome and leading cause of maternal mortality in the region. We recommend larger and even multi-centre national studies to further confirm our findings and investigate predictors of mortality for HDP so as to reduce the resultant maternal mortality.

## Conclusion

Our findings suggest an increasing trend in ICU admissions for HDP with high but stable mortality rate. Most patients present to health facility in advanced disease states with target organ damage. Interventions to strengthen and improve antenatal care services as well as multidisciplinary management approach may play a crucial role in reducing the burden of HDP in Cameroon.
